# Supplementary material for: MetaRibo-Seq measures translation in microbiomes
Source: Nat Commun. 2020 Jun 29;11:3268. doi: 10.1038/s41467-020-17081-z (PMC7324362; doi:10.1038/s41467-020-17081-z)
Supplement: Supplementary file 10 — Supplementary Data 7 [file 41467_2020_17081_MOESM10_ESM.zip › File2/Confidence_High_Taxonomy/433176_out.krona.html]

Javascript must be enabled to view this page.

members
magnitude
magnitudeUnassigned
count
unassigned
taxon
rank

433176\_out

109

2
superkingdom
109

phylum
109
976

200643
109
class


SRS019381\_contig\_number\_11243SRS053335\_contig\_number\_14774SRS077086\_contig\_number\_14039SRS893256\_contig\_number\_2260
4
order
171549
109

2005525
family
1

375288
genus
1


SRS053356\_contig\_number\_46218
species
1
46503

2005519
1
family

397864
1
genus


SRS105153\_contig\_number\_16091
1
species
397865

family
2
171550

genus
2
239759

species
1
28117

SRS893342\_contig\_number\_3786

1
species
328814

SRS148511\_contig\_number\_13103

family
101
815


SRS015854\_contig\_number\_13594SRS019030\_contig\_number\_9300SRS1041164\_contig\_number\_contig-100\_1746.39994SRS144362\_contig\_number\_13795SRS893378\_contig\_number\_4709
genus
5
101
816


SRS018836\_contig\_number\_23266
species
1
290053

29523
species
1

SRS147425\_contig\_number\_8686

1739517
1
species

SRS077552\_contig\_number\_16073

469590
4
species

SRS013215\_contig\_number\_361SRS013965\_contig\_number\_41865SRS017307\_contig\_number\_22348SRS024549\_contig\_number\_91


SRS013382\_contig\_number\_531SRS014235\_contig\_number\_28803SRS014855\_contig\_number\_5089SRS014923\_contig\_number\_16820SRS015579\_contig\_number\_21029SRS017521\_contig\_number\_18079SRS019178\_contig\_number\_2984SRS019267\_contig\_number\_132SRS020394\_contig\_number\_12001SRS022071\_contig\_number\_38510SRS044535\_contig\_number\_9285SRS049402\_contig\_number\_2038SRS062427\_contig\_number\_6624SRS062701\_contig\_number\_16SRS074964\_contig\_number\_1058SRS100021\_contig\_number\_2941SRS1041090\_contig\_number\_5596SRS1041134\_contig\_number\_1730SRS1041146\_contig\_number\_74SRS104975\_contig\_number\_20739SRS144603\_contig\_number\_17796SRS145497\_contig\_number\_19992SRS147272\_contig\_number\_2136SRS147346\_contig\_number\_contig-100\_1162.344408SRS147614\_contig\_number\_9308SRS893253\_contig\_number\_136SRS893279\_contig\_number\_1356SRS893380\_contig\_number\_155SRS893382\_contig\_number\_1139
29
species
817

species
1
457387

SRS1055076\_contig\_number\_11444


SRS1055083\_contig\_number\_3100SRS143417\_contig\_number\_1900SRS143895\_contig\_number\_48719
1946089
3
species

46506
2
species

SRS064645\_contig\_number\_1511SRS1041132\_contig\_number\_8550

47
species
820

SRS012273\_contig\_number\_41514SRS013158\_contig\_number\_4744SRS013800\_contig\_number\_17744SRS015217\_contig\_number\_contig-100\_1259.186489SRS016056\_contig\_number\_8153SRS016840\_contig\_number\_2425SRS017247\_contig\_number\_511SRS019808\_contig\_number\_9790SRS020508\_contig\_number\_216SRS024075\_contig\_number\_148SRS024435\_contig\_number\_809SRS024435\_contig\_number\_35190SRS042284\_contig\_number\_5467SRS043667\_contig\_number\_484SRS046068\_contig\_number\_2610SRS048164\_contig\_number\_8160SRS049959\_contig\_number\_46530SRS050520\_contig\_number\_86SRS050925\_contig\_number\_14683SRS050941\_contig\_number\_21592SRS051610\_contig\_number\_1554SRS051882\_contig\_number\_18580SRS053573\_contig\_number\_3385SRS056537\_contig\_number\_38929SRS063813\_contig\_number\_5303SRS063928\_contig\_number\_715SRS065397\_contig\_number\_5962SRS074586\_contig\_number\_5029SRS075773\_contig\_number\_38554SRS076804\_contig\_number\_12895SRS077294\_contig\_number\_17909SRS077502\_contig\_number\_6522SRS1041129\_contig\_number\_11686SRS1041130\_contig\_number\_1717SRS104311\_contig\_number\_7633SRS104912\_contig\_number\_18634SRS1055022\_contig\_number\_19195SRS1055069\_contig\_number\_13777SRS143085\_contig\_number\_4379SRS143991\_contig\_number\_15006SRS144753\_contig\_number\_12507SRS146764\_contig\_number\_27329SRS146813\_contig\_number\_5055SRS148091\_contig\_number\_9203SRS148253\_contig\_number\_4545SRS893270\_contig\_number\_23192SRS893358\_contig\_number\_472

1
species
28116

SRS046712\_contig\_number\_3282

47678
1
species

SRS049773\_contig\_number\_contig-100\_864.168831


SRS020271\_contig\_number\_2156SRS056259\_contig\_number\_3885SRS098881\_contig\_number\_4015SRS145308\_contig\_number\_165
821
species
4


SRS147377\_contig\_number\_5597
1
species
310297
